# Effect of Tracheostomy on Weaning Parameters in Difficult-to-Wean Mechanically Ventilated Patients: A Prospective Observational Study

**DOI:** 10.1371/journal.pone.0138294

**Published:** 2015-09-17

**Authors:** Chor-Kuan Lim, Sheng-Yuan Ruan, Feng-Ching Lin, Chao-Ling Wu, Hou-Tai Chang, Jih-Shuin Jerng, Huey-Dong Wu, Chong-Jen Yu

**Affiliations:** 1 Department of Internal Medicine, Division of Chest Medicine, Far Eastern Memorial Hospital, New Taipei City, Taiwan (R.O.C); 2 Department of Internal Medicine, Division of Pulmonary and Critical Care Medicine, National Taiwan University Hospital, Taipei, Taiwan (R.O.C); 3 Department of Integrated Diagnostics and Therapeutics, National Taiwan University Hospital, Taipei, Taiwan (R.O.C); University of Colorado Denver, UNITED STATES

## Abstract

**Background and Objective:**

Weaning parameters are commonly measured through an endotracheal tube in mechanically ventilated patients recovering from acute respiratory failure, however this practice has rarely been evaluated in tracheostomized patients. This study aimed to investigate changes in weaning parameters measured before and after tracheostomy, and to explore whether the data measured after tracheostomy were associated with weaning outcomes in difficult-to-wean patients.

**Methods:**

In a two-year study period, we enrolled orotracheally intubated patients who were prepared for tracheostomy due to difficult weaning. Weaning parameters were measured before and after the conversion to tracheostomy and compared, and the post-tracheostomy data were tested for associations with weaning outcomes.

**Results:**

A total of 86 patients were included. After tracheostomy, maximum inspiratory pressure (mean difference (Δ) = 4.4, 95% CI, 2.7 to 6.1, *P*<0.001), maximum expiratory pressure (Δ = 5.4, 95% CI, 2.9 to 8.0, *P*<0.001) and tidal volume (Δ = 33.7, 95% CI, 9.0 to 58.5, *P*<0.008) significantly increased, and rapid shallow breathing index (Δ = -14.6, 95% CI, -25.4 to -3.7, *P*<0.009) and airway resistance (Δ = -4.9, 95% CI, -5.8 to -4.0, *P*<0.001) significantly decreased. The patients who were successfully weaned within 90 days of the initiation of mechanical ventilation had greater increments in maximum inspiratory pressure (5.9 vs. 2.4, *P* = 0.04) and maximum expiratory pressure (8.0 vs. 2.0, *P* = 0.02) after tracheostomy than those who were unsuccessfully weaned.

**Conclusions:**

In conclusion, the conversion from endotracheal tube to tracheostomy significantly improved the measured values of weaning parameters in difficult-to-wean patients who subsequently weaned successfully from the mechanical ventilator. The change was significant only for airway resistance in patients who failed weaning.

**Trial Registration:**

ClinicalTrials.gov NCT01312142

## Introduction

Liberation from mechanical ventilation (MV) is an important process in managing patients with acute respiratory failure (ARF) when their condition improves. Since most patients with ARF are ventilated through an endotracheal tube, this process typically starts by assessing the patient’s readiness to wean, followed by a spontaneous breathing trial (SBT) [1], and ends with the decision of whether or not to extubate the patient. To assess weaning readiness, a number of measurements related to the physiological aspects of respiratory mechanics and capacity are usually performed [2]. These measurements, also known as weaning parameters, commonly include the maximum inspiratory pressure (MIP), maximum expiratory pressure (MEP), tidal volume (V_T_), minute ventilation (V_E_), respiratory rate (RR), rapid shallow breathing index (RSBI), airway resistance (R_AW_) and compliance (C_RS_) [3]. These weaning parameters have been reported to serve as a criteria or consideration for readiness to wean and may be able to guide the decision to extubate among vulnerable patients who are susceptible to extubation failure [1, 4].

Tracheostomy is usually suggested for ventilator-dependent or difficult-to-wean patients [5]. Although the benefits of tracheostomy on weaning outcomes remain controversial [6], replacement of the endotracheal tube with a tracheostomy tube has been shown to reduce the work of breathing and improve the clearance of airway secretion [7–11]. To the best of our knowledge, studies regarding the effect of tracheostomy on weaning parameters are limited. Therefore, this study aimed to compare weaning parameters measured before and after the endotracheal tube was converted to tracheostomy, and to explore whether such changes were associated with weaning outcomes in difficult-to-wean patients.

## Materials and Methods

### Settings and Patients

This prospective observational study was conducted from January 2011 to December 2012 at the intensive care units (ICUs) and respiratory care center (RCC) of National Taiwan University Hospital, a tertiary referral medical center in Taiwan. Our RCC cared for mechanically ventilated patients transferred exclusively from this hospital’s ICUs for possible weaning. Patients aged > 18 years, orotracheally intubated due to acute respiratory failure, who had received spontaneous breathing trials (SBTs) but fulfilled the criteria of difficult-to-wean and consented to undergo tracheostomy, were screened for eligibility of enrollment. Based on previous consensus statement in the literature for difficult and prolonged weaning [2], we defined ‘difficult-to-wean’ in this study as either one of the two criteria: 1) patients who required at least three or more attempts of SBT but all failed; 2) patients who remained not successfully weaned after seven days since the first SBT. T-piece trial was the primary method used in the SBT. Patient who failed the initial T-piece trial might either repeat T-piece trial on next attempt or change to try other methods for SBT, such as pressure support ventilation mode (at a supporting pressure of 6 cmH_2_O) or automated tube compensation method, as determined by the attending physicians in the intensive care unit. The attending physician in the intensive care unit also determined the reason of difficult weaning by clinical judgement. Patients with the following conditions were excluded: SBT was successful but extubation was not performed subsequently; tracheostomy was not expected to be performed within 2 weeks after informed consent had been obtained; patients who remained staying in the ICU after tracheostomy without subsequent transfer to the RCC; use of a fraction of inspired oxygen of > 0.4 at a positive end-expiratory pressure of 5 cmH_2_O. The Research Ethics Committee of National Taiwan University Hospital approved the study (approval number: 201012007RC), and a written informed consent was obtained from each patient or the surrogate before enrollment.

### Tracheostomy and Weaning After the Conversion

We used surgical instead of percutaneous method for tracheostomy in our hospital. The operating surgeon for tracheostomy determined the size of the tracheostomy tube. The tracheostomy tube we used was non-fenestrated Hi-Lo tracheostomy tube with cuff (Shiley^TM^, Covidien, Mansfield, MA, USA). After the post-operative condition was stabilized, the patient was transferred to the RCC unless the attending physician indicated the need for extended stay in the ICU before the transfer. Once the clinical condition was considered stable, the primary care team reduced the settings of mechanical ventilation and started the weaning process based on a standardized protocol for weaning used in the ICUs and RCC of this hospital. The method for SBT involved the provision of oxygen via a T-tube while the patient was breathing spontaneously without being connected to the ventilator, while the duration of SBT was determined on a daily basis according to the protocol. The attending physician determined the timing of initiating SBT, and the respiratory therapist adjusted the duration of daily SBT until the patient became able to tolerate the whole-day trial of spontaneous breathing. Weaning success was defined as the liberation from ventilator support for at least 5 consecutive days, whereas weaning failure with SBT was judged based on the physiologic criteria defined as previously reported [2]. If a patient failed an SBT, the patient would be rested with full ventilator support for the rest of the 24 hours, and followed by a stepwise reduction of MV support on the next day until the patient became ready for SBT again, and this process usually took at least 24 hours, but the physician might resume SBT earlier if it took short period for stepwise reduction of MV support. If the second SBT remained unsuccessful, the attending physician would determine the timing and method for subsequent weaning attempts. The patients who were successfully weaned were transferred to the general wards before they were discharged from the hospital. For the patients who failed weaning would be transferred out of the RCC within 6 weeks of stay, either to the general ward of this hospital, or to another hospital with facilities for long-term ventilator care, and the attending physician in the RCC assessed the reason of weaning failure.

### Measurement of Weaning Parameters

The weaning parameters, including MIP, MEP, RR, spontaneous V_T_, spontaneous V_E_, RSBI, C_RS_, and R_AW_, were measured within 24 hours before the conversion, and within 24 hours after the conversion to tracheostomy, by the respiratory therapist who participated in the study, using a standardized method as previously described [12]. The two participating respiratory therapists had a working experience of more than 15 years involving ventilator management and weaning. Briefly, the therapist placed the patient in a semi-Fowler position before the measurement, checked the tracheostomy tube to ensure adequate inflation of the cuff balloon, and cleared the airway secretion by suctioning. The therapist then disconnected the endotracheal or tracheostomy tube from the ventilator, and connected the tube to a spirometer (Wright Respirometer, Haloscale Standard, nSpire Health Ltd, Longmont, CO, USA). After the respiratory movements of the patient became steady, the therapist calculated the RR by observing movements of the thorax and abdomen, recorded the V_E_ by taking the measured scale on the spirometer at the end of one minute, and calculated the V_T_ by dividing V_E_ by RR, as well as the RSBI by dividing RR by V_T_. To measure the MIP and MEP, the therapist connected the endotracheal or tracheostomy tube with a T-tube, placed a manometer (Boehringer Laboratories, Norristown, PA, USA) to one end of the T-tube via a nipple adaptor and a unidirectional valve to the other end. After the inspiratory port of the unidirectional valve was manually occluded, the therapist coached the patient to inhale actively against the occluded airway during the breathing cycles. The test lasted for 20 to 25 seconds, and the therapist took the most negative value read from the pointer as the MIP. A total of three valid tests were performed, and the highest MIP value was used for analysis [12]. The MEP was measured in a similar manner to that of MIP except that the expiratory port of the unidirectional valve was manually occluded. To measure R_AW_ and C_RS_, the ventilator was set to the volume-control mode, with the airflow waveform set as square, the flow at 60 L/min, the tidal volume at 8–10 mL per kg of predicted body weight, and the inspiratory hold at 0.2–0.5 seconds. With the peak and plateau pressures measured during inspiratory hold, R_AW_ was calculated as the difference between these pressures, divided by inspiratory flow. C_RS_ was calculated as the set tidal volume divided by the difference between plateau and end-expiratory pressures.

### Data Collection and Analysis

We collected clinical and demographic data including age, gender, co-morbidities, admission diagnosis, reason for intubation, results of weaning trials after tracheostomy, dates of endotracheal intubation and conversion to tracheostomy, and status of mechanical ventilation upon leaving the RCC. The primary aim of the study was to compare the weaning parameters before and after the conversion to tracheostomy. The secondary aim was to evaluate the associations of weaning parameters as well as their changes with weaning success at 90 days after the initiation of MV.

### Statistical Analysis

The values of clinical data and weaning parameters were expressed as mean ± SD. The paired *t*-test or Wilcoxon signed-rank test was used to compare the values of weaning parameters measured before and after conversion to tracheostomy, as appropriately. The independent *t*-test or Mann-Whitney U test was used to compare changes in weaning parameters between patients who were successfully and unsuccessfully weaned, as appropriately. Pearson’s χ^2^ test or Fisher’s exact test was used to compare categorical variables between weaning success and weaning failure group, as appropriately. We used receiver operating characteristic (ROC) curve to find out the cut-off value of post-tracheostomy weaning parameters. An area under curve (AUC) that greater than 0.6 was considered appropriate. Logistic regression model was used to measure the predicting factors for successful weaning after tracheostomy. Variables with a p-value of < 0.05 in the univariate analysis were selected to determine the final model. A p-value of < 0.05 was considered statistically significant. All the statistical analyses were performed with SPSS software (version 22 for Mac) (SPSS Inc., Chicago, IL, USA).

## Results

**Fig 1:**
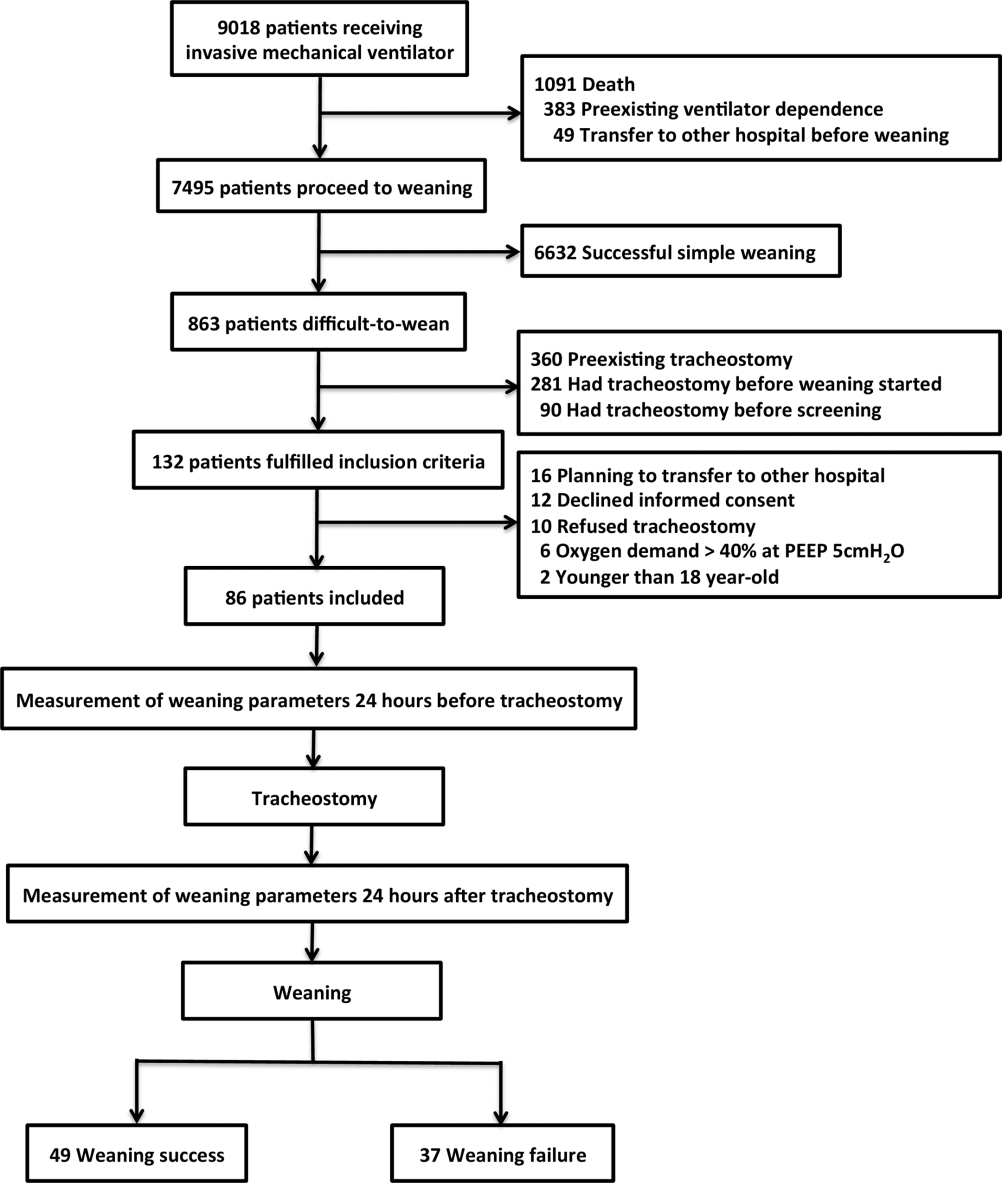
The flow chart of this study.

Fig 1 demonstrates the flow chart of our study. A total of 86 patients with ARF and difficult weaning were included, of whom 59% were male, and 66% (n = 57) were older than 65 years. Their clinical and demographic characteristics are summarized in Table 1. Diabetes mellitus (32%) was the most common comorbidity, and pneumonia (42%) was the most common reason of ARF. These patients had a mean duration of 20 days of MV use before tracheostomy, and upon enrollment, 66 (77%) of them had a body mass index between 18 and 30. The reasons of difficult weaning, as judged clinically by the attending intensivists, included poor endurance in 37 (43.0%) patients, poor cough power in 16 (18.6%) patients, poor airway hygiene in 16 (18.6%) patients, poor respiratory compliance in 9 (10.5%) patients and poor cardiac function in 8 (9.3%) patients. None of the patients had a replacement of the endotracheal tube with a new one before the conversion to tracheostomy.

**Table 1:** Clinical characteristics of 86 mechanically ventilated difficult-to-wean patients included in this study.

Characteristic	Patients Number	Weaning Success	Weaning Failure	*p* value*
Total number	86	49	37	
Age	70 ± 16.7	68 ± 17.8	72 ± 15.1	0.40
Male	51 (59%)	26 (51%)	25 (49%)	0.18
Body mass index	22.3 ± 4.2	22 ± 4.6	22 ± 3.7	0.82
Reason of Intubation				
Pneumonia	36 (42%)	23 (64%)	13 (36%)	0.27
Surgery	16 (19%)	13 (81%)	3 (19%)	0.03
Hypercapnic Respiratory Failure	14 (17%)	4 (29%)	10 (71%)	0.02
Airway Protection	6 (7%)	4 (67%)	2 (33%)	0.62
Heart Failure	4 (5%)	0 (0%)	4 (100%)	0.02
Pulmonary Hemorrhage	3 (4%)	2 (67%)	1 (33%)	0.73
Cardiac Arrest	3 (4%)	2 (67%)	1 (33%)	0.73
Sepsis	2 (2%)	1 (50%)	1 (50%)	0.84
Myocardial Infarction	2 (2%)	0 (0%)	2 (100%)	0.10
Comorbidities				
Diabetes mellitus	27 (31%)	17 (63%)	10 (37%)	0.58
Malignancy	24 (28%)	13 (57%)	11 (43%)	0.74
Congestive heart failure	19 (22%)	6 (32%)	13 (68%)	0.01
Chronic obstructive pulmonary disease	15 (17%)	6 (40%)	9 (60%)	0.15
Size of tracheostomy tube¶				
Larger	53 (62%)	26 (49%)	27 (51%)	0.06
Similar or smaller	33 (38%)	23 (70%)	10 (30%)	
Ventilator days before tracheostomy	20 ± 12.2	19 ± 11	21 ± 14	0.38

* Comparison between weaning success and weaning failure, by Pearson’s χ^2^ test or Fisher’s exact test, as appropriately.

¶ Comparison with the size of endotracheal tube before tracheostomy.

Almost all (98%) of the patients had an endotracheal tube with an inner diameter of either 7 mm (34 patients) or 7.5 mm (50 patients), which was replaced by a tracheostomy tube with an inner diameter of either 7 mm (32 patients) or 8 mm (52 patients). In one patient, the size of the endotracheal tube was 6.5 mm, which was replaced by a 7-mm tracheostomy tube. Another one patient had an 8-mm endotracheal tube replaced by an 8-mm tracheostomy tube. Of all 86 patients, 53 (62%) had their endotracheal tube replaced by a tracheostomy tube with a larger inner diameter, whereas 27 (31%) with a same-diameter one and only 6 (7%) with a smaller one.

Table 2 summarizes the data and comparisons of the measured values of weaning parameters for the 86 patients before and after the conversion to tracheostomy. Before tracheostomy, 68 (79%) patients had a MIP higher than 20 cmH_2_O, and this increased to 76 (88%) after tracheostomy. The number of patients with an MEP higher than 20 cmH_2_O also increased from 60 (70%) to 67 (78%), while patients with RSBI lower than 105 also increased from 53 (62%) to 60 (70%). After tracheostomy, 45 (52%) had an increased MIP, 46 (53%) had an increased MEP, and 46 (53%) had an increased V_T_, whereas 52 (60%) had a decreased RSBI, and 69 (86%) had a decreased R_AW_. As shown in Table 2, the mean MIP, MEP and V_T_ values increased significantly after tracheostomy, whereas RSBI and R_AW_ decreased significantly.

**Table 2:** Weaning parameters measured before and after tracheostomy.

Weaning Parameters	Before Tracheostomy (mean ± SD)	After Tracheostomy (mean ± SD)	Difference (mean ± SD)	*p* value*
MIP, cmH_2_O	32.0 ± 11.5	36.4 ± 11.8	4.41 ± 8.1	< 0.001
MEP, cmH_2_O	30.8 ± 13.9	36.3 ± 16.2	5.43 ± 11.8	< 0.001
V_T_, mL	294.5 ± 114.2	328.2 ± 122.3	33.73 ± 115.4	0.008^a^
V_E_, L/min	7.4 ± 3.4	7.7 ± 3.2	0.34 ± 2.7	0.24
RR, frequency/min	25.5 ± 7.0	24.5 ± 6.7	-0.98 ± 6.5	0.17
RSBI	102.0 ± 53.0	87.4 ± 43.3	-14.6 ± 50.5	0.019
C_RS_, mL/cmH_2_O	34.0 ± 12.7	35.8 ± 12.3	1.8 ± 9.5	0.09^a^
R_AW_, cmH_2_O·s/L	18.0 ± 6.2	13.2 ± 5.9	-4.9 ± 4.5	< 0.001

MIP: maximum inspiratory pressure; MEP: maximum expiratory pressure; V_T_: tidal volume; V_E_: minute ventilation; RR; respiratory rate; RSBI: rapid shallow breathing index; C_RS_: compliance; R_AW_: airway resistance.

* Comparison of weaning parameters before and after tracheostomy, by paired t test or Wilcoxon signed-rank test, as appropriately.

^a^ By paired t test

Of the 86 patients, 49 (57%) were successfully weaned. They had a mean MV duration of 38.4 days (range, 12–89 days), mean duration of in-hospital stay after tracheostomy of 71 days (range, 13–231 days), and a mean in-hospital length of stay of 102 days (range, 44–245 days). Forty-five of them survived to hospital discharge without resuming MV. Of the 37 (43%) patients who failed weaning from the ventilator within 90 days of MV, 30 were alive at hospital discharge, and only one patient was successfully weaned after 90 days of MV. These 37 patients who failed weaning had an average hospitalization of 47 days after tracheostomy (range, 5–177 days), with a total hospitalization of 78 days in average (range, 22–201 days). The mortality rate of the 86 patients was 13% (11 patients) at hospital discharge.

Table 3 compares the measured values of weaning parameters before and after tracheostomy in these patients, separated by the weaning status at 90 days after MV initiation. In the patients who were successfully weaned, we found that the majority of the measured values of the parameters, including MIP, MEP, V_T_, V_E_ and R_AW_, improved significantly after the conversion to tracheostomy. In the patients who failed weaning, in contrast, only R_AW_ improved significantly. Table 4 compared these two groups of patients in the extent of changes in weaning parameters after the conversion to tracheostomy. The patients who were successfully weaned had greater increments of MIP and MEP after tracheostomy than those who failed weaning. We also compared the before-after changes of measured values of weaning parameters in the 53 patients who had a tracheostomy tube with a larger inner diameter than their original endotracheal tube, and the 33 whose tracheostomy tube size not larger that the original endotracheal tube. We found that the changes were similar in these two groups in terms of changes in MIP (p = 0.43), MEP (p = 0.81), V_T_ (p = 0.87), V_E_ (p = 0.37), RSBI (p = 0.41), R_AW_ (p = 0.24) and C_RS_ (p = 0.48).

**Table 3:** Comparison of weaning parameters measured before and after tracheostomy in patients with weaning success and weaning failure.

Weaning Parameters	Weaning Success (n = 49)			Weaning Failure (n = 37)		
	Before	After	*p* value*	Before	After	*p* value*
MIP	33.4 ± 10.3	39.4 ± 11.2	< 0.001^a^	30.0 ± 12.9	32.4 ± 11.6	0.057
MEP	31.6 ± 14.1	39.6 ± 14.7	< 0.001	29.9 ± 13. 7	31.9 ± 17.2	0.32
RR	25.1 ± 6. 7	24.9 ± 7.2	0.88	26.1 ± 7.4	24.0 ± 6.1	0.080
V_T_	313 ± 106	361 ± 123	0.028	270 ± 122	284 ± 108	0.35
V_E_	7.5 ± 2.8	8.3 ± 3.1	0.040	7.2 ± 4.1	6.9 ± 3.2	0.53
RSBI	93.0 ± 51.3	79.1 ± 41.3	0.15	114 ± 53	98 ± 44	0.063
C_RS_	33.9 ± 11.8	36.2 ± 11.8	0.056^a^	34.2 ± 14.0	35.3 ± 13.0	0.56
R_AW_	18.5 ± 6.2	13.2 ± 5.8	< 0.001	17.5 ± 6.2	13.1 ± 6.0	< 0.001^b^

MIP: maximum inspiratory pressure; MEP: maximum expiratory pressure; V_T_: tidal volume; V_E_: minute ventilation; RR; respiratory rate; RSBI: rapid shallow breathing index; C_RS_: compliance; R_AW_: airway resistance.

* Comparison of weaning parameters before and after tracheostomy, by paired t test or Wilcoxon signed-rank test, as appropriately.

^a^ By paired t test.

^b^ By Wilcoxon signed-rank test.

**Table 4:** Comparison of the changes in weaning parameters in patients with different weaning outcome and patients with different tracheostomy tube size.

Weaning Parameters	Difference of measured data (After—Before)			Difference of measured data (After—Before)		
	Weaning Success (n = 47)	Weaning Failure (n = 37)	*p* value*	Larger Diameter¶ (n = 53)	Similar or Smaller Diameter¶ (n = 33)	*p* value*
ΔMIP, cm_H_2_O	5.92 ± 8.24	2.41 ± 7.44	0.04	4.83 ± 7.75	3.73 ± 8.60	0.43
ΔMEP, cm_H_2_O	8.02 ± 11.16	2.00 ± 11.94	0.02	5.68 ± 10.39	5.03 ± 13.98	0.81
ΔV_T_, mL	47.98 ± 128.09	14.86 ± 94.52	0.19^c^	35.41 ± 122.28	31.03 ± 105.24	0.87^c^
ΔV_E_, L/min	0.81 ± 2.56	-0.279 ± 2.67	0.06^c^	0.543 ± 2.67	-0.279 ± 2.67	0.37^c^
ΔRR, frequency/min	-0.12 ± 5.86	-2.11 ± 7.11	0.16	0.11 ± 6.37	0.016 ± 2.63	0.03
ΔRSBI	-13.86 ± 52.04	-15.50 ± 49.12	0.88^c^	-11.02 ± 48.82	-20.25 ± 53.40	0.41^c^
ΔC_RS_, mL/cmH_2_O	2.28 ± 8.15	1.08 ± 11.07	0.56	2.41 ± 10.30	0.72 ± 8.0	0.48
ΔR_AW_, cmH_2_O·s/L	-5.24 ± 4.32	-4.41 ± 4.42	0.38	-4.62 ± 4.82	-5.30 ± 3.51	0.24

MIP: maximum inspiratory pressure; MEP: maximum expiratory pressure; V_T_: tidal volume; V_E_: minute ventilation; RR; respiratory rate; RSBI: rapid shallow breathing index; C_RS_: compliance; R_AW_: airway resistance.

* Comparison of weaning parameters between weaning success and weaning failure, by independent t test or Mann-Whitney U test, as appropriately.

^c^ By independent t test.

¶ Larger diameter indicates the size of tracheostomy tube was larger than the endotracheal tube, similar or smaller diameter indicates the size of tracheostomy tube was similar or smaller than the endotracheal tube.

**Fig 2:**
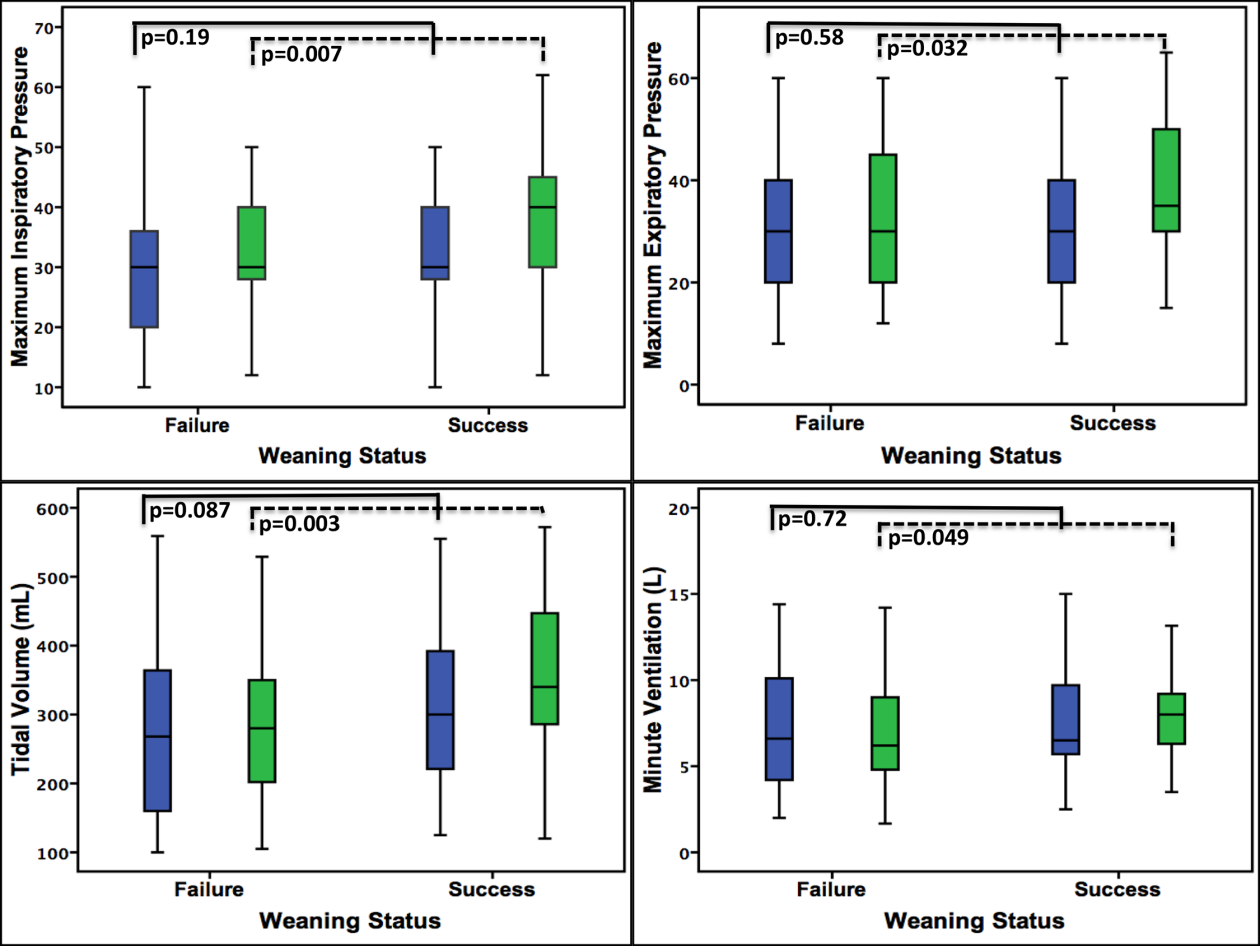
Comparison of weaning parameters between weaning success and weaning failure group. Before tracheostomy, the weaning parameters were no difference between the two groups. After tracheostomy, the MIP, MEP, Vt, Ve and RSBI showed significant improvement in the weaning success group.

**Fig 3:**
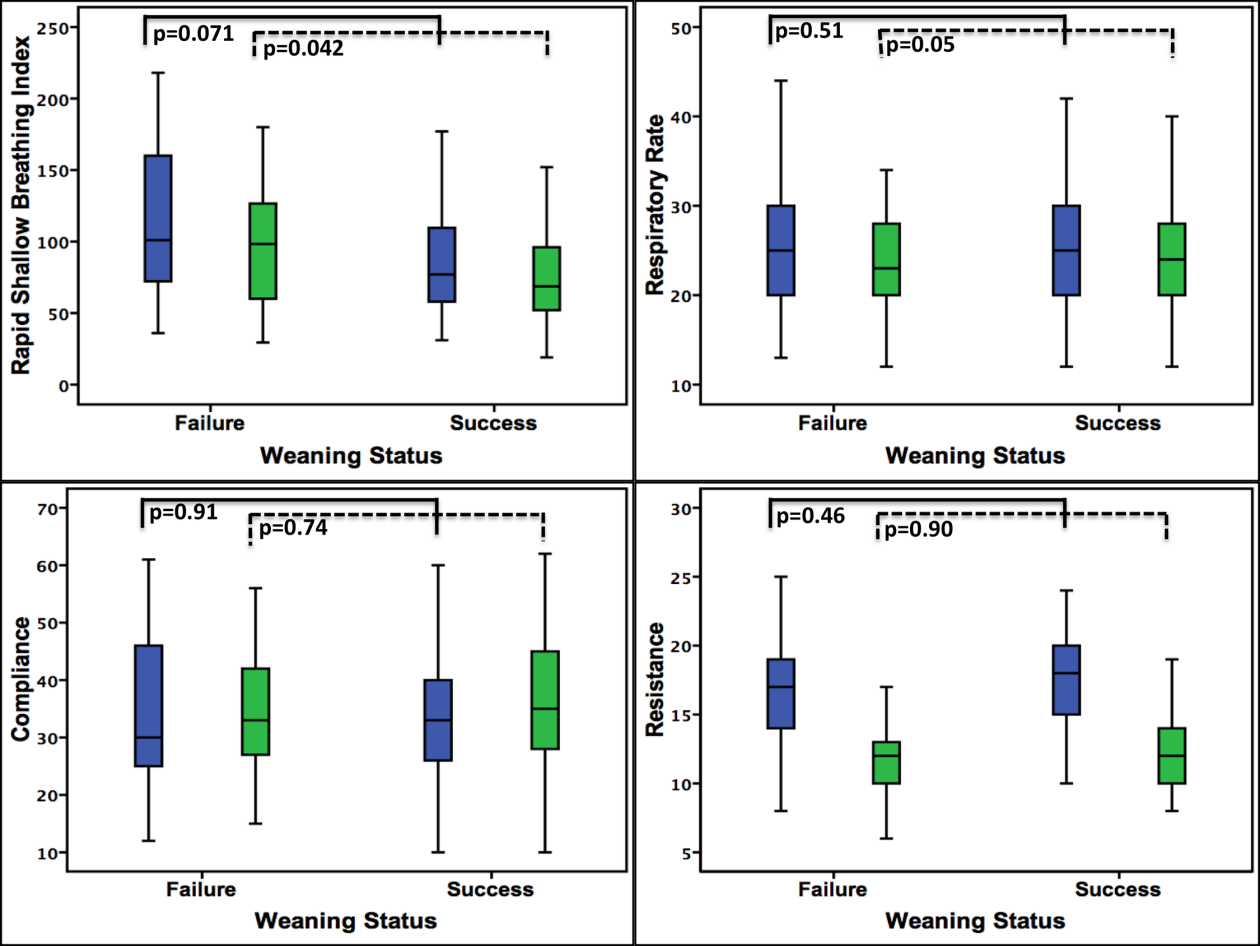
Comparison of weaning parameters between weaning success and weaning failure group. Before tracheostomy, the weaning parameters were no difference between the two groups. After tracheostomy, the MIP, MEP, Vt, Ve and RSBI showed significant improvement in the weaning success group.

Figs 2 and 3 also showed the comparison of weaning parameters between the patients with weaning success and weaning failure. Before the conversion to tracheostomy, the measured values of weaning parameters between these two groups were all similar, after tracheostomy, however, the MIP (p = 0.007), MEP (p = 0.032), V_T_ (p = 0.003), V_E_ (p = 0.049) and RSBI (p = 0.042) were significantly better in the weaning success group than the weaning failure group.

We used the receiver operating characteristic curve to find the cut-off values of post-tracheostomy weaning parameters. We did not find an appropriate cut-off for RR (AUC:0.48; p = 0.72), C_RS_ (AUC:0.54; p = 0.56) and R_AW_ (AUC:0.46; p = 0.54). Nonetheless, potential cut-offs were found for MIP (AUC:0.68; p = 0.005; cut-off:33; sensitivity:69%; specificity:65%; PPV:0.72; NPV:0.62), MEP (AUC:0.63; p = 0.04; cut-off:31; sensitivity:63%; specificity:51%; PPV:0.63; NPV:0.51), V_T_ (AUC:0.69; p = 0.003; cut-off:285mL; sensitivity:76%; specificity:60%; PPV:0.71; NPV:0.65), V_E_ (AUC:0.63; p = 0.036; cut-off:6.7L; sensitivity:71%; specificity:54%; PPV:0.67; NPV:0.59) and RSBI (AUC:0.64; p = 0.03; cut-off:93; sensitivity:71%; specificity:57%; PPV:0.69; NPV:0.60).

By multivariate logistic regression, we found that the statistically significant independent predictors of weaning success in these difficult-to-wean patients who received tracheostomy included post-tracheostomy MIP < -33 (OR:3.30; 95% CI:1.20–9.17; p = 0.02), V_T_ ≥ 285mL (OR:3.20; 95% CI:1.15–8.87; p = 0.03) and patients with congestive heart failure (OR:0.23; 95% CI:0.07–0.76; p = 0.02), as shown in Table 5.

**Table 5:** Multivariate analysis of factors for predicting weaning success in 86 difficult-to-wean patients who received tracheostomy.

Factors	Univariate Analysis P value	Multivariate Analysis P value	OR (95% CI)
Gender, man vs. woman	0.18		
Age, > 70 vs. < 70	0.87		
Reason of Intubation			
Hypercapnia, yes vs. no	0.02	0.14	
Surgery, yes vs. no	0.03	0.10	
Heart failure, yes vs. no	0.02	0.27	
Comorbidities			
Congestive heart failure, yes vs. no	0.01	0.02	0.23 (0.07–0.76)
Tracheostomy tube size¶			
Larger vs. similar or smaller	0.06		
Post-tracheostomy weaning parameters			
MIP, cmH_2_O	0.002	0.02	
≥ -33			1
< -33			3.30 (1.20–9.17)
MEP, cmH_2_O	0.18	0.60	
< 31			
≥ 31			
V_T_, mL	0.001	0.03	
< 285			1
≥ 285			3.20 (1.15–8.87)
V_E_, L	0.02	0.48	
< 6.7			
≥ 6.7			
RSBI	0.008	0.97	
< 93			
≥ 93			
Ventilator days before tracheostomy	0.38		

MIP: maximum inspiratory pressure; MEP: maximum expiratory pressure; V_T_: tidal volume; V_E_: minute ventilation; RSBI: rapid shallow breathing index; OR: odd ratio; CI: confidence interval.

¶ Comparison with the size of endotracheal tube before tracheostomy

## Discussion

In this study we found that in difficult-to-wean patients, the weaning parameters measured via the tracheostomy tubes differed substantially from those measured via the endotracheal tubes on the same patient, and that the changes were more significant among the patients who weaned successfully after their conversion to tracheostomy. While weaning parameters have been widely applied to evaluate the probability of weaning success in patients who are intubated orotracheally [13–16], our findings also suggest the potential of applying post-tracheostomy weaning parameters to evaluate the prognosis of weaning.

The differences in weaning parameters measured via the endotracheal and tracheostomy tubes may be due to a number of factors related to the nature of the tubes and the patients. The obvious contrast in length and shape between the two types of tubes may explain the expected difference in the effect on airway resistance. It has previously been shown that tracheostomy tubes decrease airway resistance by an average of 3 cm H_2_O compared to orotracheal tubes [8]. In the real-world clinical settings, however, the difference of resistance might be even more significant, because in most cases of difficult weaning, an endotracheal tube typically had been placed for a prolonged duration that coating of the airway secretions on the inner wall of tube lumen might further increase the resistance during breathing. Furthermore, any physical change of the endotracheal tube, such as episodes of kinking and damage to the inner wall by repeated suctioning, might further cause an impact on tube resistance. In this study, the patients had been orotracheally intubated for an average of 20 days without tube replacement; this might explain our finding that the mean difference in airway resistance came as high as 4.9 cmH_2_O, and might at least partly explain the beneficial effect of conversion to tracheostomy from orotracheal intubation. We also found that the MIP and MEP increased after the conversion to tracheostomy. As C_RS_ remained unchanged after tracheostomy in the patients, the increase in MIP and MEP might be attributed to the improved comfort of the patients after tracheostomy as speculated in the literature [17], which might make the forceful inspiration and expiration easier to perform during the measurement.

Our finding that patients who weaned successfully had greater improvements in weaning parameters after tracheostomy than those who failed weaning suggests that at least some group of patients might benefit from the conversion from endotracheal tube to tracheostomy. While reports in the literature showed a reduction in work of breathing after tracheostomy [9] and suggested improved airway suctioning efficiency [17], we also found a significant increase of tidal volumes postoperatively. The two sessions of measurements of parameters were carried out at an interval of no longer than 48 hours as described in the Methods, typically no longer than 24 hours, it was unlikely that the increase in tidal volume was due to improved strength of the respiratory muscles. A previous study on 18 surgical patients and 2 patients with aspiration pneumonia reported no significant increases in tidal volume after tracheostomy [8]. Our different findings may be explained by that while surgical patients in the previous study tend to have better respiratory muscle strength when weaning is initiated than those intubated for pneumonia, 52.4% of the patients in our study had the comorbidity of chronic cardiopulmonary diseases. Similar to our findings, in the study of Diehl et al,[9] which enrolled difficult-to-wean patients, the V_T_ after tracheostomy also tended to increase. The duration of endotracheal intubation might again play a major role that the probability of thick coating with airway secretion in the endotracheal tube would be resolved by the conversion to tracheostomy when the measurement of the parameters was performed shortly after the operation.

V_E_ showed a significant increment in weaning success group after tracheostomy. While respiratory rate remained unchanged after tracheostomy, the increment of V_E_ might be attributed to the significant improvement V_T_ after tracheostomy in the weaning success group, which might be explained by an improved MIP. Meanwhile, the weaning parameters between weaning success and weaning failure group were no difference before tracheostomy. Nevertheless, MIP, MEP, V_T_, V_E_ and RSBI improved significantly in the weaning success group after tracheostomy, as compared to the weaning failure group. Our result implied a possible prognostic value of post-tracheostomy weaning parameters.

In the clinical settings, accurate measurement of the weaning parameters almost always depends on the skills of the respiratory therapist, and therefore might be affected by the variations in measurement. By standardizing the measuring technique, nevertheless, it has been reported that weaning parameters could be reliably measured at bedside [18]. To our knowledge, there has been a scarcity of reports in the literature assessing the reliability of weaning parameter measurement in patients with tracheostomy. The two respiratory therapists participating in this study had a long experience of working in this field, and they applied the same standardized methods of measurement as was commonly used in our institution, therefore we believe that this potential practice which might serve as a guide to clinical care, and warrant further prospective investigation in a larger scale.

There are a number of limitations to this study. First, conducted at a single-center with a relatively small number of patients, this study has provided novel findings, but prospective studies at a larger scale are needed to test the generalizability. Second, this study only focused on the difficult-to-wean patients in whom extubation was not attempted because of failed attempts of SBT; our findings therefore may not be applied to patients with extubation failure. Third, 62% of the patients in this study had a their endotracheal tubes converted to a tracheostomy tube with a larger inner diameter; this might overestimate the contribution of the tube conversion alone to the improvement of weaning parameters, despite that our study also reflected the choice for larger diameter tube for tracheostomy in the real-world practice. Fourth, for the measurement of MIP and MEP in this study, the methods applied were isovolumetric, which was performed in a duration of less than 5 minutes; we therefore could not exclude the possibility of a learning effect when the measurement was repeated after tracheostomy, which was at an interval of no longer than 48 hours. Fifth, the demography between weaning success and weaning failure group were not totally comparable. These might have some impact on the weaning outcome; however, they did no affect the measurement of post-tracheostomy weaning parameters and the changes of these parameters after tracheostomy. Sixth, the AUC of the ROC curve for MIP, MEP, V_T_, V_E_ and RSBI ranged between 0.6 to 0.7, which is not considered as a satisfactory cut-off, despite that the MIP and V_T_ after tracheostomy are significantly associated with weaning success after the conversion to tracheostomy. This might be attributed to the small patient number in this study. We hope that this study may act as the pebble that makes the ripple and encourage more effort in the future to explore the significance of post-tracheostomy weaning parameters. Seventh, this study was conducted when our knowledge of the best practice for the weaning method in tracheostomised patients with prolonged MV had remained undetermined. As Jubran A et al had later demonstrated that unassisted breathing through tracheostomy had a shorter weaning duration in tracheostomized patients with prolonged MV [19], further studies might be needed to guarantee our findings. Nevertheless, since this study did not involve the randomization of different interventions, we believe the weaning method might play a less important role in the interpretation of our findings

## Conclusions

In conclusion, the conversion from endotracheal tube to tracheostomy significantly improved the measured values of weaning parameters in difficult-to-wean patients who subsequently weaned successfully from the mechanical ventilator. The change was significant only for airway resistance in patients who failed weaning.

## Supporting Information

S1 Dataset Dataset of the study population. Dataset file includes detailed information which is collected and analyzed in this study.
(XLS)

## Abbreviations

ARF: Acute respiratory failure
C_RS_: Compliance
ICU: Intensive care unit
MEP: Maximum expiratory pressure
MIP: Maximum inspiratory pressure
MV: Mechanical ventilation
R_AW_: Airway resistance
RCC: Respiratory care center
RR: Respiratory rate
RSBI: Rapid shallow breathing index
SBT: Spontaneous breathing trial
VE: Minute ventilation
V_T_: Tidal volume
PPV: Positive predictive value
NPV: Negative predictive value
